# Context and determinants for implementing a sepsis survivor care transition intervention reported from five health systems and home health agencies

**DOI:** 10.3389/fmed.2025.1632083

**Published:** 2025-12-01

**Authors:** Kathryn H. Bowles, Michael A. Stawnychy, Melissa O'Connor, Mark E. Mikkelsen, Nancy Hodgson, Elaine Sang, Sang Bin You, Katherine Pitcher, Jiyoun Song, Sungho Oh, Brittany Newman, Patrik Garren, Charlotte Weiss, Karen B. Hirschman

**Affiliations:** 1 Department of Biobehavioral Health Science, NewCourtland Center for Transitions & Health, University of Pennsylvania School of Nursing, Philadelphia, PA, United States; 2 Center for Home Care Policy & Research, VNS Health, New York City, New York, United States; 3 Leonard Davis Institute of Health Economics, University of Pennsylvania, Philadelphia, PA, United States; 4 Hunter-Bellevue School of Nursing, New York City, New York, United States; 5 University of Colorado Anschutz Medical Campus, Aurora, CO, United States; 6 College of Arts and Sciences, University of Pennsylvania, Philadelphia, PA, United States

**Keywords:** sepsis, implementation science, care transitions, barriers & facilitative factors, patient transfer, home health care (HHC), ICD-10

## Abstract

**Introduction:**

Care transitions from acute to post-acute care are complex, especially for sepsis survivors. Implementation science offers valuable insights to translate best practices and improve care transitions. Our objective is to explore the context (site characteristics and personnel) and determinants (barriers, proposed strategies, and facilitators) influencing I-TRANSFER, a Type 1 hybrid implementation science study aimed at providing timely home health and outpatient visits for sepsis survivors within 1 week of hospital discharge.

**Methods:**

Qualitative, descriptive design with interviews guided by the eight study objectives and the Consolidated Framework for Implementation Research. Ninety-one leaders in clinical, quality, and administrative roles caring for sepsis survivors in five healthcare systems (16 hospitals) and five affiliated home health care agencies in four states participated. Deductive and inductive thematic analysis of 61 interviews conducted using NVivo 14. Proposed strategies were mapped to the Expert Recommendations for Implementing Change (ERIC) taxonomy.

**Results:**

A total of 32 themes emerged. Barriers included *care coordination, staffing, electronic health record (EHR), information transfer,* and *access to care.* Informants proposed ERIC strategies to address barriers such as *changing record systems, facilitating relay of clinical data to providers, conducting education meetings, or revising professional roles.* Facilitators occurred across several themes: *EHR; information transfer; staffing;* c*are coordination; access to care; home health policies, pathways, and processes; and quality monitoring.*

**Conclusion:**

The interviews produced actionable insights for leaders, clinicians, providers, and policy makers regarding identifying sepsis through clear definitions, using the problem list and ICD-10 coding. Scheduling outpatient care, communicating to the next level of care, and providing timely follow-up and care coordination necessitates attention to staffing, tools for scheduling and quality measurement, and EHR integration for information transfer. Patient education is critical for awareness of risk and informed decision-making regarding follow-up after discharge.

## 1 Introduction

The evidence base for effective care transitions post-hospital discharge is well established (1, 2), yet there is limited knowledge about implementation barriers (3, 4), facilitators, or strategies (3, 5). Evidence-based care transition interventions ensure optimal continuity of care for patients moving between healthcare settings (1). However, the current lack of knowledge about the context and determinants of successful care transition implementation can lead to repeated errors, wasted resources and time, and inconsistent implementation quality (6). Communicating barriers and facilitators is essential for advancing implementation science and translating evidence to practice (7).

Care transitions are inherently complex and fraught with challenges such as fragmented (1) or delayed care (8), poor communication, and insufficient information exchange (9). The period immediately following hospital discharge is critical, especially for high-risk conditions like sepsis (10). Sepsis patients experience life-threatening organ dysfunction caused by a dysregulated immune response to infection (11). While improved recognition and rapid treatment have increased survival rates (12), sepsis is still the leading cause of 30-day readmission (13) and survivors frequently experience post-sepsis syndrome (14, 15), reinfection (16), and exacerbation of chronic conditions (10). Among sepsis survivors transitioned to home health care (HHC), 32% of 30-day readmissions occurred within the first week (17), underscoring the need for implementation of evidence-based care transition protocols.

The I-TRANSFER (Improving TRansitions ANd OutcomeS oF SEpsis SuRvivors) implementation science study (2R01NR016014) examines the implementation and effectiveness of a best practice protocol to reduce 30-day readmissions for sepsis survivors (18). The evidence-based protocol, developed in a previous comparative effectiveness study (19), involves a HHC start-of-care nursing visit within 2 days of hospital discharge, an additional nursing visit, and an outpatient provider visit within the first week post-discharge. Sepsis survivors who received this protocol had 30-day readmission rates 7% lower than their counterparts; a 41% relative reduction (19). However, only 28% of sepsis survivors nationwide received this pattern of care (19).

Prior to I-TRANSFER implementation, a needs assessment was conducted to assess the context and determinants of implementation. Here, we report our findings and discuss them in the context of other care transition implementations, the Centers for Disease Control (CDC) Hospital Sepsis Program Core Elements (20) and provide implications for practice, research, and policy.

## 2 Materials and methods

### 2.1 Study design

**Figure 1:**
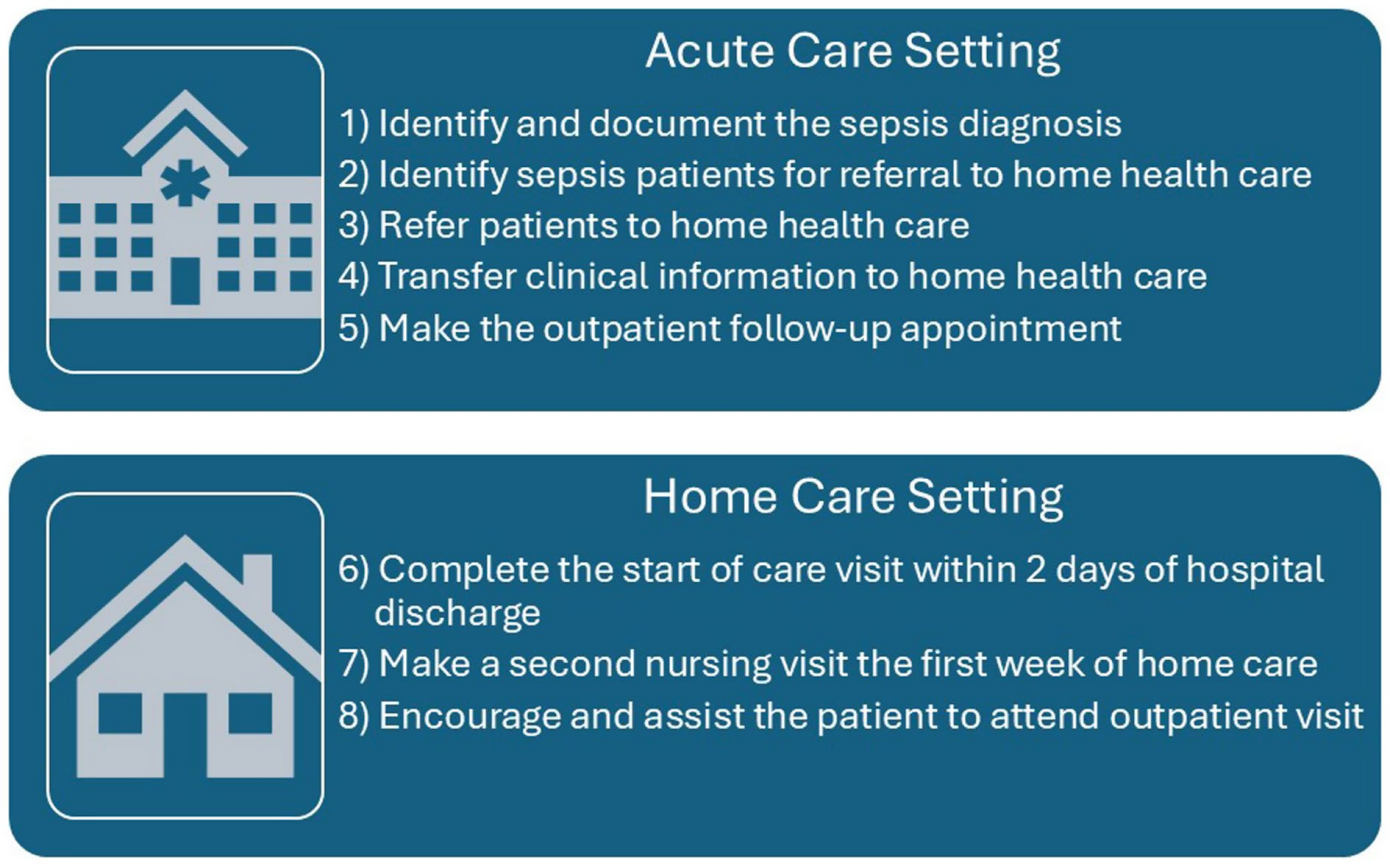
Objectives for I-TRANSFER implementation by health care setting.

We used a qualitative descriptive design with individual and group needs assessment interviews guided by the eight objectives (obj) of the I-TRANSFER protocol (Figure 1). Interviews also considered the five domains of the Consolidated Framework for Implementation Research (CFIR): Innovation, Outer Setting, Inner Setting, Individuals, and the Implementation Process (21), and context-site characteristics, roles, and circumstances (22). Determinants include barriers that hinder achieving protocol objectives, proposed strategies to overcome them, and facilitators that support implementation (22). Proposed strategies were organized using the Expert Recommendations for Implementing Change (ERIC) taxonomy (23).

### 2.2 Setting and subjects

To support transferability (24), sites were purposefully selected for variation in size, location, and integration (hospital and HHC under the same system). Leaders in clinical, quality, or administrative roles from five healthcare systems and five affiliated HHC agencies (“dyads”) were recruited by the Principal Investigator (PI) (KHB). Site leaders identified a purposeful sample (25) of individuals involved in sepsis care and transitions to HHC and outpatient care. Chain-referral sampling was used to recruit additional informants (26).

The study was approved by two Institutional Review Boards (University of Pennsylvania School of Nursing and VNS Health) and informants provided verbal consent. Methods and results are reported in accordance with the COnsolidated criteria for REporting Qualitative research (COREQ) checklist (27) (Supplementary Table 1).

### 2.3 Data collection

Following consent, (KHB, MOC, and MS) conducted 60-min, semi-structured Zoom interviews between May and November 2021 with one additional group interview in June 2022 when one hospital joined later. The interview guide was published previously (28). Interviews were recorded, transcribed, cleaned and de-identified by trained research assistants, and uploaded to NVivo 14 for analysis (29). Field notes captured context and role descriptions. Data saturation was reached when context and process were fully described.

### 2.4 Data analysis

The analytic team included the PI (KHB), three Co-Investigators (MOC, NH, KBH), postdoctoral fellows (MS, SO, CW) and pre-doctoral students (ES, MGT). Research team members trained together applying thematic analysis (30) to create an initial codebook and deductively code four random transcripts, achieving 90% agreement. Independent coding followed, with 20% of interviews double coded for intercoder reliability (range 91–97%). Weekly meetings refined the codebook. Inductive thematic analysis coded the barriers and facilitators into themes. The proposed strategies were mapped to the ERIC taxonomy (23) by (CW) and confirmed by consensus with (KHB and MOC). Findings were reported back to the informants for member-checking (credibility) (31). Detailed notes and site/informant characteristics enhanced transferability (31, 32). Results were discussed with our National Advisory Committee, comprised of experts in sepsis and HHC, to increase credibility and transferability (31).

## 3 Results

### 3.1 Context- informants

A total of 91 informants participated in 61 interviews: 36 individual and 25 group interviews with 57 informants from acute care and 34 from HHC or outpatient care. Informants’ roles included administrators, care and intake coordinators, hospitalist and intensivist physicians, advanced practice nurses, quality managers and coders, clinicians with roles in direct care delivery, and sepsis coordinators.

### 3.2 Context- site characteristics

The I-TRANSFER study includes five health systems with 16 hospitals, paired with five HHC agencies (five dyads) in four states. Nine hospitals are in large academic medical centers (dyads 1, 4, 5), seven are community-based (dyads 2, 3) with medium and small bed sizes, respectively. Three HHC agencies (dyads 1–3) are owned by the partnering health system (integrated); two are independent (dyads 4, 5). Table 1 provides descriptives of the informants and sites.

**Table 1:** Characteristics of informants and sites.

Characteristics	Categories	*n* (%) informants
Sex^*^	Female	60 (70.6)
Race^*^	White	69 (82.1)
	Asian	9 (10.7)
	Black or African American	1 (1.2)
	Native Hawaiian or Other Pacific Islander	1 (1.2)
	Chose not to answer	4 (3.8)
Ethnicity^*^	Non-Hispanic	78 (92.9)
	Hispanic or Latino	2 (2.4)
	Not reported	4 (4.7)
Sites	Dyad 1 (6 hospitals) with one integrated HHC agency (Academic medical center, ≥425 beds, urban, Northeastern US, integrated EHR)	39 (42.9)
	Dyad 2 (4 hospitals) with one integrated HHC agency (Community, 250–424 beds, urban, Northeastern US, non-integrated EHR)	16 (17.6)
	Dyad 3 (3 hospitals) with one integrated HHC agency (Community, <250 beds, urban, Western US, non-integrated EHR)	7 (7.7)
	Dyad 4 (2 hospitals) with one independent HHC agency (Academic medical center, ≥425 beds, urban, Northeastern US, non-integrated EHR)	21 (23.1)
	Dyad 5 (1 hospital) with one independent, for-profit HHC agency (Academic medical center, ≥425 beds, urban, Mountain US, integrated EHR)	8 (8.8)
Setting	Acute Hospital informants	57 (62.6)
	Home health or outpatient informants	34 (37.4)
Role	Administrators	19 (20.9%)
	Care and intake coordinators	19 (20.9%)
	Hospitalists/intensivists	18 (19.8%)
	Advance practice nurses	12 (13.2%)
	Quality managers and coders	10 (11%)
	Care delivery: Registered nurse, social worker, physical therapist, and occupational therapist	9 (9.9)
	Sepsis coordinators	4 (4.4%)

^*^N = 84; 91 stakeholders participated in the interviews; 84 reported their demographics.

### 3.3 Themes

**Figure 2:**
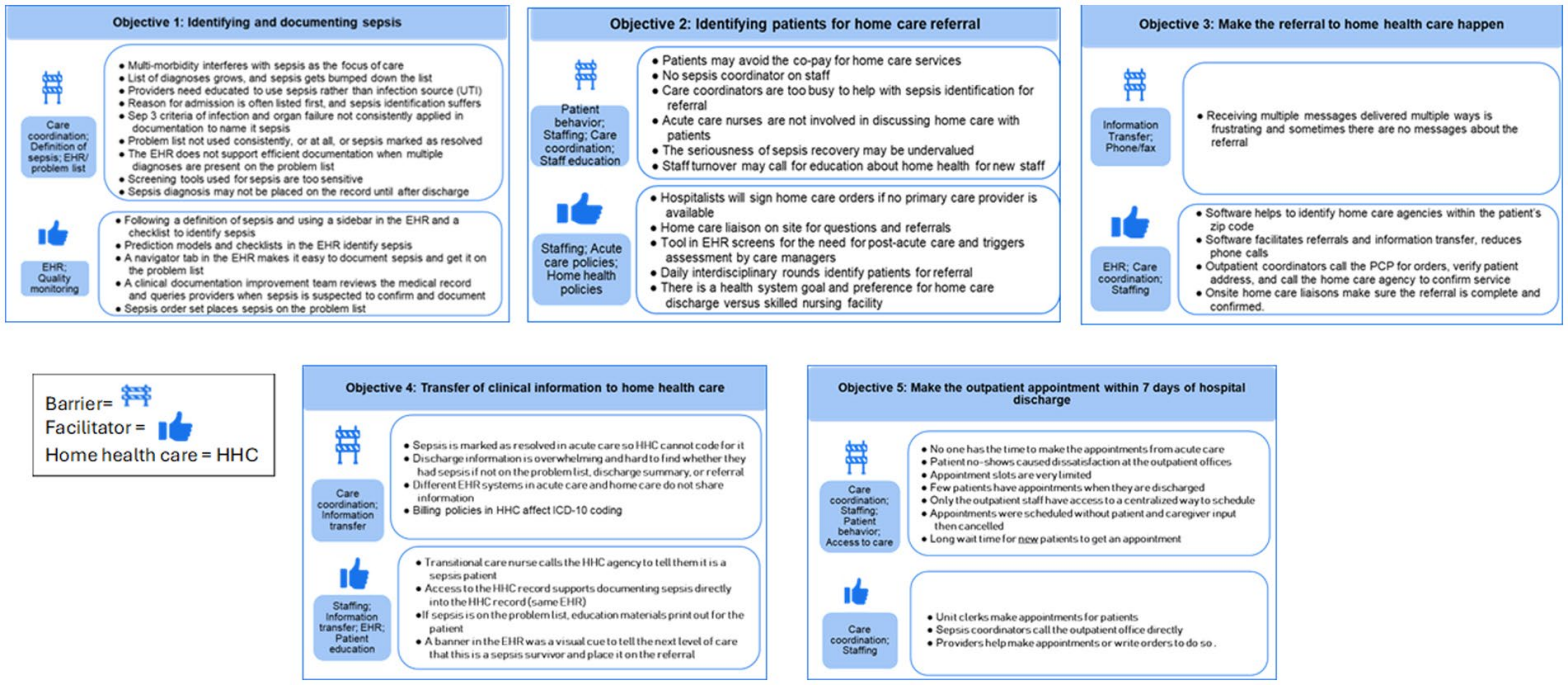
Barrier and facilitator themes and examples by I-TRANSFER objectives 1–5 (acute care).

**Figure 3:**
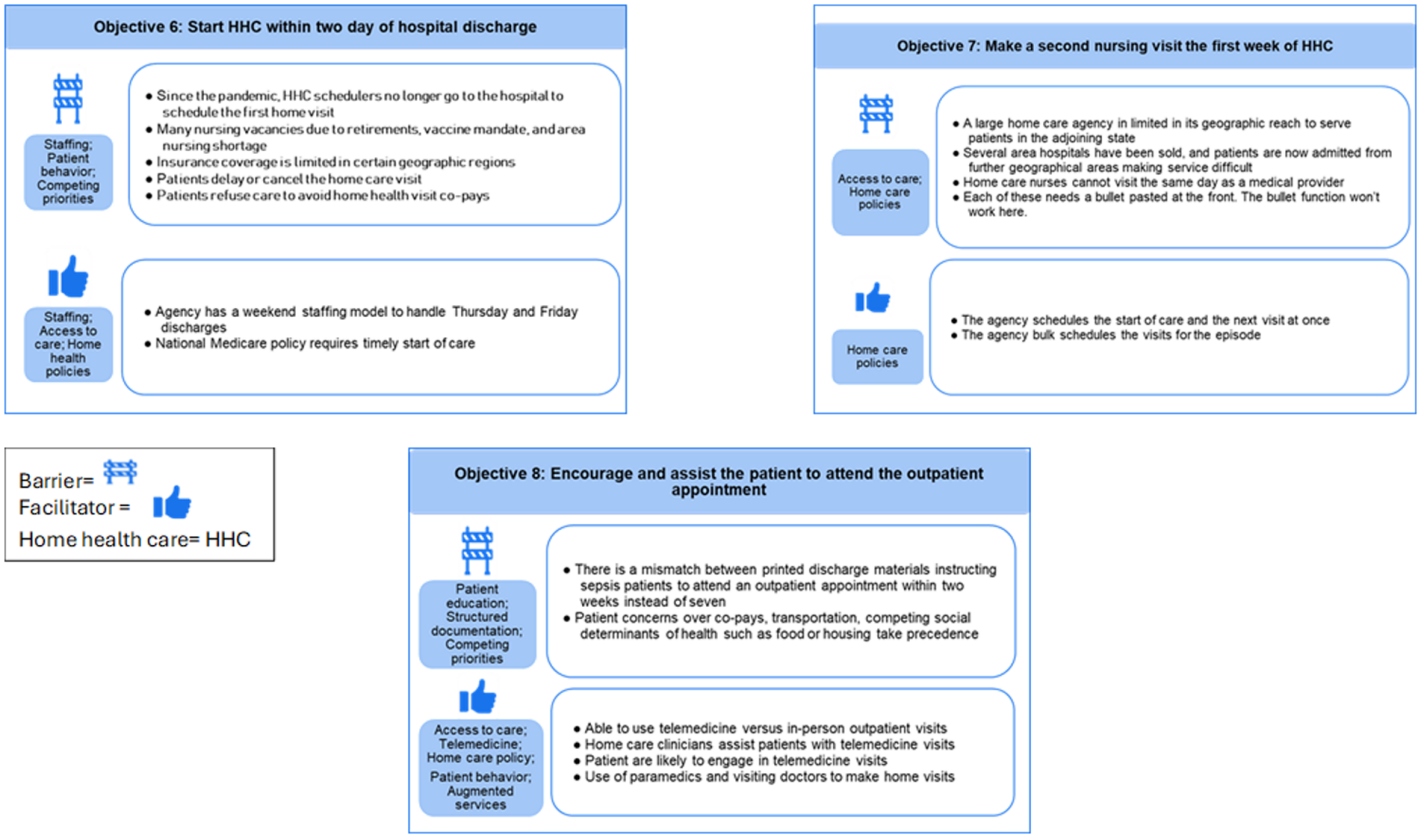
Barrier and facilitator themes and examples by I-TRANSFER objectives 6–8 (home health and outpatient).

Qualitative coding of the barriers, proposed strategies, and facilitators discussed in the interviews resulted in 32 themes listed and defined in Table 2. Below, we indicate *themes* and *(subthemes)* in italics reported as barriers and facilitators. Figures 2, 3 provide summaries of the barriers and facilitators for each objective. Exemplary quotes illustrating barriers and facilitators are provided in Table 3 for acute care objectives 1–5 and Table 4 for HHC objectives 6–8. To protect confidentiality, quotes are labeled by the site of the informant (e.g., acute care) rather than the role of the person since some sites had one person in a particular role (e.g., sepsis coordinator).

**Table 2:** Themes, subthemes, and definitions.

Themes	Subthemes	Definitions
Care coordination		Deliberate and organized patient care activities between and across care settings as part of standard practice/processes.
	Scheduling	The act of scheduling appointments for patient care.
Staffing		The availability and/or functions of staff involved in acute care and post-acute care of sepsis survivors.
Electronic health record (EHR)		Information technology for the collection, storage, and reporting of clinical and administrative health-related patient data.
	EHR alert	A flag, banner, or alert in the EHR to call attention to information or send a reminder.
	Use of the problem list	The way the problem list is used (or not used) to communicate the sepsis diagnosis in the electronic health record.
	Structured documentation	Information captured electronically in standardized fields (e.g., drop down menus).
	Unstructured documentation	Any free text or images in documentation that would require qualitative review or natural language processing to extract.
Information transfer		The capability to move information among different healthcare settings or levels of care.
	Phone-Fax	Use of the telephone or fax machine for communication.
	Messaging system	Information transfer within a secure system allowing for text messaging between or within an organization.
Access to care		Physical, tangible items determining access to health care that are constant and exist physically. These can be improved through care coordination.
	Augmented services	Additional services, beyond traditional to assist patients to obtain care.
	Financial insurance	Health insurance and the financial situation of the patient
	Geographic location	Geographic location of where the patient resides that impacts service areas of home health care or outpatient visit attendance.
	Outpatient provider	The presence or involvement of a primary care or specialist provider in the outpatient setting which may influence timely evaluation and management after discharge.
	Transportation	Transportation availability to outpatient appointments.
	Telemedicine	The use of telecommunication technologies to deliver healthcare services virtually.
Definition of sepsis		Criteria used by providers to diagnose sepsis.
Provider or staff behavior, decisions, and preferences		Healthcare providers and staff actions affecting patient care.
Patient education		Providing information to impact general health and/or sepsis-related knowledge, attitudes, and skills for self-management.
Competing priorities		When multiple needs, obligations, or responsibilities conflict with one another, requiring individuals to make trade-offs.
	Social determinants of health	Conditions in the places where people live, learn, work, and play that affect a wide range of health and quality-of-life risks and outcomes.
Patient behavior, decisions, and preferences		Patient actions that affect their healthcare.
	No-show outpatient	Patient did not attend the scheduled appointment.
	Decline or delay home health	Patients chose to modify, not to initiate, or postpone the recommended services.
Home health agency policies, pathways, and processes		Established guidelines, procedures, and workflows that govern how care is initiated, delivered, and managed within the home health care organization.
Quality monitoring of service		The process of evaluating care delivery to identify issues, implement improvement initiatives, and track their effectiveness over time to ensure optimal outcomes.
Informal caregiving		A family member or friend who is involved in the patient’s care.
Acute care policies, pathways, and processes		Established guidelines, procedures, and workflows that govern how sepsis care is initiated, delivered, and managed within the acute care organization.
External policies and incentives		A broad construct that includes external strategies to spread interventions, including policy and regulations (governmental or other central entity), external mandates, recommendations and guidelines, pay-for-performance, collaboratives, and public or benchmark reporting.
Staff education		The provision of information and training to acute and post-acute healthcare staff about sepsis care and related activities.

**Table 3:** Exemplary quotes for I-TRANSFER objectives in acute and outpatient care.

Objective 1: Identifying and documenting the sepsis diagnosis
Care coordination - Barriers
“Yeah, I mean I think the hard part is, number one, the identification … you know, if their diagnosis inpatient is sepsis, does that then correlate to a discharge of sepsis because we know a lot of these people have a lot of other comorbidities. They come in as a UTI, their CHF gets in the way, and then we all get jumbled in what our focus of care is…so we do not even know about them until they are already on their way home, and I think that that’s a challenge.” (acute care, dyad 2)
“I’m not sure diagnosis in general or sepsis is adequately communicated. I think we have a working DRG that pops up in- we have a column in our electronic health record for a working DRG, which is you know a moving target. I think there’s competing diagnoses. Sometimes if someone’s here and they are listed as sepsis, they have a procedure or something else crops up it bumps sepsis lower on the list. So, [for] I think identification of the patient, I think there’s probably opportunity to improve it.” (acute care, dyad 1)
“A lot of times, whoever, and some of this is really just playing with ongoing physician education and provider education is to try to use sepsis as the diagnosis it that’s really the main diagnosis because we often see that a patient might be listed as UTI or pyelonephritis or pneumonia, bacteremia instead of really having that keyword sepsis.” (acute care, dyad 2)
“I was just looking at many charts that I knew had sepsis and the identification just wasn’t there. Because that’s not what the problem was. The patient came in with lower back pain and they ended up having sepsis due to hardware. So up front the sepsis identification was not there.” (acute care, dyad 5)
Definition of sepsis - Barrier
“It would align with the sepsis three [Sep 3] definition and it would acknowledge the clinician’s documentation failure, which is quite common that we as clinicians recognize infection, we separately recognized organ failure, but often in the heat of the moment of a daily medical record do not call it sepsis. The usual approach would be someone writes pneumonia and then at some point they A navigator tab write acute kidney injury, but they do not link them in some clear and consistent and transparent sort of documentation method.” (acute care, dyad 1)
EHR - use of the problem list - Barrier
“So, the problem list is usually very off track. Sometimes it says no problems and the patient has been here for 10 days. Sometimes it says epistasis and the patient is here for AKA. It’s just not accurate.” (acute care, dyad 5)
“…you know-[EHR brand], which we use, is definitely designed to run from documentation from problem lists. Our system chose not to use that approach, and it’s for good reasons. It’s bulky, it’s- it’s hard to categorize which problem is more important. So, we do not document in the problem list in [EHR brand]…So, if I come in with sepsis, but then I also have COPD and they are both high, where do they line up? …and looking at the note, it looks ugly. And that’s why people are hesitant to use that. But that would solve most of these problems because you are forced to identify it as sepsis or not. But we do not document that way. That could be- if we were forced to use that-probably solves about 80% of the problem.” (acute care, dyad 1)
“Something we have done is to add that sepsis diagnosis in to [an] order set to go on the problem list. So that’s kind of how we identify it. We do know that we are not that great at- from a problem list standpoint, we are not that great because we miss it on both sides. The screening, I would call it, is very sensitive for sepsis. Some people we name sepsis end up not being coded for sepsis. And then on the other side, sometimes, even though we are treating appropriately as in the documentation ends up being coded for sepsis, it’s not on the problem list. So, we are not that great from a problem- problem list to call it sepsis. We missed it on both sides. Although, we are much better at documenting. So, in the notes … we are very good at calling it sepsis in our notes. That’s how it gets coded. But from a problem list in [EHR brand] standpoint, a lot of things rely on that. We’re, you know, we are actually better than most of the places. We’re still not that good.” (acute care, dyad 1)
“I cannot tell you the number of times I open a new H&P [history and physical] and there are no problems documented in the problem list, and so I have to take it on myself to actually, actively document, some of the things that are going on with that patient. This patient has sepsis, this patient has hyponatremia, for example, and I try to document those things for the record, not just for us, but, as we all know, for continuity sake, for the next provider of care whether that’s a home health care person, primary care doctor or another healthcare facility, if that patient gets readmitted.” (acute care, dyad 1)
EHR - Facilitators
“So, we have a definition of sepsis that we follow. And we have some predictive models and EHR that suggest that a patient may have sepsis. And it currently- it’s basically based on SIRS [systemic inflammatory response syndrome], etc. And in [EHR brand], we have a sidebar and a checklist that kind of says ‘This patient may have sepsis’. And it starts early in the emergency department” (acute care, dyad 1).
“It did take some time to actually get this as a checklist within [EHR brand] but I’m hopeful that it will kind of target providers to thinking more about sepsis. Um and it does have a whole navigator tab where they can do certain documentation, as well as have access to the problem list to hopefully add sepsis to the problem list earlier on, because I think that’s the easiest way really to sort through [EHR brand] if Sepsis is not on the problem list. It’s very easy to generate a list of patients from the start.” (acute care, dyad 2)
Quality monitoring of service - Facilitator
“The way that this is handled in the back end is through what’s known as CDI team, the clinical documentation improvement team. We recognize that the inability to disseminate the information up front is a challenge, there will be a slow uptake and then the CDI team emails clinicians in real time, and both educates them and prompts them to use different terminology so there’s the back-end way to address this.” (acute care, dyad 1)
Objective 2: Identify sepsis patients for home health referral
Patient behavior, decisions, and preferences - Barrier
“Sometimes patients have co-pays and they do not want to pay. That’s a big barrier, the financial part.” (acute care, dyad 1)
Staffing - Barrier
“We do not have an identified individual who is the sepsis coordinator and down the line I think if work like this wants to be sustained and continued, I think that is a key role and kind of figuring out how to demonstrate the importance of that role not only qualitatively, but fiscally I guess to the institution is going to be something that we are going to have to think about.” (acute care, dyad 1)
“I have not had luck with the care coordinators, which is the reason why we went to the directors of med surg. I try [tried] to work with care coordination, so I just work around them. They were so busy.” (outpatient, dyad 3)
Care coordination - Barrier
“Education is provided to patients, but I would say that the amount of knowledge set of [acute care] nurses with home care agencies is little to none, and they, they are not really involved in that discharge process in terms of discussing home care or discussing sepsis at home, I would say.” (acute care, dyad 4)
Staff education - Barrier
“A lot of times, things have a lot of emphasis and then the emphasis moves, and so I actually think that the sepsis alert and just the whole sepsis issue, really, probably is kind of under-valued at this point. For example, in acute care we do some work with a lot of new nurses, and I’m really curious actually if they even understand what the narrator is, if they are ever formally trained on it, or if it’s assumed that the preceptor will discuss it. I’m actually wondering if my nurses even know what to do with the narrator. I actually do not think they do, to be honest.” (acute care, dyad 4)
“We’re seeing a lot of case managers leaving the case management world. So, I’m sure that the people who have been there, a long time, and that they know the drill, they probably are pushing that for the patients, but maybe some of the newer hired case managers just really have not had time to learn that is part of their role, so I’m sure it just varies case manager to case manager.” (acute care, dyad 5)
Staffing - Facilitators
“…initially we’d have the hospitalist sign the home care orders. And they usually will do that in the interim.” (acute care, dyad 1)
“If there’s a barrier or question, [they have] an on-site liaison here at our hospital for about four hours each day, Monday through Friday. He’s also available through Microsoft Teams that we can message, or he has a phone that he carries. So, we are in close contact with him throughout the day for any of those patients that are questionable or we are trying to work together to get services.” (acute care, dyad 1)
Acute care policies, pathways, and processes - Facilitator
“…it’s a tool that we use that’s in [EHR brand]. And it asks several different questions. We actually just did some recent enhancements to it. But it’s asking things like, what risk factors does the patient have at home? Do they live alone? Do they have support? How was their daily function? It kind of goes over all those areas to help us determine if they are going to have discharge needs. And that’s done just by chart review. And if they, what we call “Screen Positive” in any of those areas, then we have a nurse care manager or a social worker see that patient face to face. And at that point they do a full evaluation and create a discharge plan.” (acute care, dyad 5)
“The process by which they get referred to home health, I mean there’s- we have an interdisciplinary team meeting every day. We call it ‘care progression rounds’ in our hospitals. Care coordination is part of that, nursing is part of that, therapy is part of that. Occasionally, you have a provider, not occasionally probably 60% of the time, you have some sort of provider that’s part of that process. And we are reviewing the status of the patient, we are identifying what their needs are post discharge, and we are making decisions based on conversations we have with the family, with the patient. Whether there’s somebody that’s going home and is going to require home care, whether they are- or whether they need some other level of care. Once they have been identified as a potential home care candidate, we make a referral to our home care agency where their liaisons come in and do an assessment of the patient and helps us with the process from there.” (acute care, dyad 2)
Home health internal policies, processes, pathways - Facilitator
“For the appropriate patients we do try to get patients home as opposed to skilled nursing facilities. You know, I think we try to be judicious with this. I think there is a push to avoid going to nursing home if they can be taken care of well at home… And that’s one of our system goals and entity goals.” (acute care, dyad 1)
Objective 3: Refer patients to home health care
Information Transfer - Barrier
“I think on our staff’s end, our staff gets really frustrated because sometimes they get multiple messages- the same message four different ways. And sometimes then- sometimes I think we do not get any messages. So that’s kind of frustrating too” (acute care, dyad 1).
EHR - Facilitators
“It [software] helps me connect my patient in a county area with a home care agency that services their specific zip code. So, I will say it worked. It got me a home care agency.” (acute care, dyad 1)
“We use [EHR brand] with [HHC agency] so all our referrals come through as [EHR brand] alerts and requests. Discharge planning software called [software brand] facilitates referrals and information transfer. It’s been a big reduction in phone calls and voicemails if everyone keeps [software brand] up and running live.” (acute care and home health, dyad 1)
Care coordination - Facilitators
“Before we even call that patient, we will call the PCP and verify that the information is actually accurate, the appointment is there. We will call the home health agency and make sure that (A) they are aware that they were referred to them and then (B) we verify they have the orders, etc., … so there’s a certain level of follow up and accountability, with the referred agencies.” (outpatient, dyad 3)
Staffing - Facilitator
“We have our [HHC agency name] liaisons that are on site that work very closely, certainly with the care coordination team, as well as the multidisciplinary team, and they represent home health, infusion, hospice, and palliative care, and so very easily if they are told, then they will go ahead and do all the legwork to make sure the referral is pretty, packaged in a bow, and sent off and accepted and taken care of.” (home health, dyad 3)
Objective 4: Transfer clinical information to home health care
Care coordination - Barrier
“I think that if it’s resolved, they will not code it for sepsis.” (acute care, dyad 5)
Information transfer - Barriers
“We’ll also be looking at discharge information, but as anybody who’s looked at discharge information has seen it’s a ton of information. So, it makes it a lot harder to sift through if you are trying to determine if somebody is a sepsis survivor by looking at a hospital chart. It’s a lot harder to see that than if it just goes right into a referral.” (home health, dyad 1)
“I really do not think it’s continued after it’s been resolved, so if the patient is not actively in sepsis, you know, is [not] actively septic any longer, then I do not believe so, it would be more just communicated in the report and notes, and things of that nature, so it would just be part of the clinical understanding.” (acute care, dyad 4)
“But if you do not share the same chart system, if you do not have access to [EHR brand], then those folks do not know that that patient was even admitted and what they were admitted for. I will say like the hospital has been a challenge in that way sometimes for some patients where, you know, they were not even aware of one of my patient’s admissions from like a year ago. And so, I had to re- resend them records…this was relevant to this patient care. And you are the primary office. And either you are not believing what a patient is telling you, or you are not requesting records from us. And this is a problem.” (acute care, dyad 1).
“You know not only can we not code it, but now our coding, you know we switched the way we code in home health for I think it was October of last year. it’s trickier to have it in our ICD 10 codes.” (home health, dyad 3)
Staffing/Information transfer - Facilitator
“When reviewing the clinical chart and the clinician is going out, they’ll be made aware. The only other stop gap would be is if we are identifying that this patient has sepsis, we being [the] transitional care center, is verifying that, “oh, look, it’s on our sepsis query. They’re a sepsis patient.” When we call the home health agency to instruct them like, “hey we are just double checking, you know, you have these orders,” as well. We do verbalize that to intake, and it is, of course, on to intake to document that internally and take it from there. With [HHC agency name], certainly, it’s an internal system. It’s a little bit easier, I can go into their chart and document that myself for them, and they can see it, but more often than not, it’s verbalized to the partner companies.” (outpatient, dyad 3)
EHR - Facilitators
“But I know as far as patients that have that sepsis diagnosis in their problem list, … when we print out their discharge paperwork it gives them a sepsis awareness list of signs and symptoms to look for. “If you run into these symptoms, please contact your doctor” or “If so severe, go back to the emergency room” (acute care, dyad 1)
“The goal of the banner was just a visual cue for my team for both case management and social work so that when they did the handoff whether it was to home care or the skilled facility they were reminded to tell the next level of care that this was a sepsis survivor and put on the referral.” (acute care, dyad 1)
Objective 5: Making the outpatient appointment within 7 days of hospital discharge
Care Coordination - Barriers
“And it’s very hard when we discharge patients to get follow-up appointments. If we can do it and it’s hard, it’s 10 times harder for patients to get through to the call centers and everything. So, you know, ideally if we could have a centralized way to do this it would be lovely. But no one has the time to do it. Unit coordinators complain, social workers cannot do it, physicians and providers cannot do it. Nobody has the time.” (acute care, dyad 1)
“I know my social work colleagues have heard me say this for years, we do not make appointments for patients when they are leaving. I think that is one key, and this has been studied, if they have an appointment when they are being discharged, they are more likely to go and see the patient- their primary care doc.” (acute care, dyad 1)
“I believe that at our hospital when we- when they took on that initiative, it was done by the unit techs. They would schedule the appointments prior to a patient’s discharge. But it just ended up being that oftentimes the patient had to reschedule the appointment anyway because it did not work. The date and time did not work for them or what have you. So, I think it just ended up being it wasn’t worth the time to do that because it wasn’t working for the patient.” (acute care, dyad 2)
“It is the ambulatory team today that has that direct access [for scheduling]. So, while we have the inpatient team trying to make that appointment, on behalf of the patient, they still have to do it through the ambulatory team. …to me that’s a barrier, we need to remove.” (outpatient, dyad 2)
“My most disheartening was I was setting up a patient with the health clinic and it was a new patient follow up and they were not accepting new patients till the year 2022. So, what they do is they would have X amount of emergent appointments every day. But as far as for a PCP appointment, this new patient 2022 I was like you have got to be kidding me.” (acute care, dyad 1)
Staffing - Barrier
“I think the concern on our end, frankly, is our staff cannot be making appointments. We do not have the bandwidth. And that’s- you know, I think everyone’s going to say that. It’s just there’s only so many of us. And to make appointments, you know?” (acute care, dyad 1)
Patient behavior, decisions, and preferences-no-show outpatient - Barrier
“The doctor’s offices were complaining because patients were not showing up for their appointments, and they were blocking all this time for hospital discharges. And then they were not making the appointments. Patients were dissatisfied because they were not getting, you know- we were booking appointments, but they did not have transportation for that time. …and then they were not necessarily taking the initiative to change the time or that kind of thing.” (acute care, dyad 1)
Care coordination - Facilitators
“We do have unit clerk secretaries on each of the units that assist with setting up follow-up appointments and placing it on the patient’s After Visit Summary. So sometimes those appointments are there, and sometimes it’s for the patient ‘please schedule an appointment with infectious disease [in] three to seven days.’ So, nursing will be able to share. I think there’s five units that are trialing that now with [what] they are doing the appointments.” (acute care, dyad 1)
Staffing - Facilitator
“I’ll reach out to that office myself and just say, you know, ‘This patient was just discharged, is required to have a post-hospital appointment with your office. Can I verify patient information with you? Can you as an outpatient provider contact this patient directly to schedule?’ And that way, it puts that onus on that provider.” (acute care, dyad 1)
“We used to have a scheduler, and they did a pilot for a year, and then they removed it, but there’s talk about having it again, where the person would just schedule appointments for them, but as of right now it’s just the physicians.” (acute care, dyad 4)

**Table 4:** Exemplary quotes for I-TRANSFER objectives in home health care.

Objective 6: Start of home health care within 2 days of hospital discharge
Staffing - Barriers
“So, we were having schedulers go to the bedside and schedule, but I think they stopped doing that with COVID. We were doing scheduling out of the hospitals and, to be honest with you, I do not know if that has returned yet.” (home health, dyad 1)
“The concerns I have is, you know, number one is resources in the outpatient side because we are struggling, as I said earlier, with getting our patients seen in a timely manner from home care because of the vacancies that they are all… well, we are all dealing with it. I mean in the hospital too. We have a bunch of vacancies for various reasons, you know, the vaccine mandate, people retiring, just the plain old shortage of people coming in, so I think that, you know, we’d have to make sure that they are going to be able to do that 48-h visit. I think that’s where I think [it] might be problematic.” (home health, dyad 2)
“Recently, it’s been staffing. Insurance is another reason. They do not service the geographic location.” (home care, dyad 1)
“Right now, particularly here, …there is a nursing shortage. It’s profound. It’s crippling.” (acute care, dyad 3)
“I’ve been in the home care arena for 30 years. The staffing challenges that I see right now are nothing that I’ve ever seen in my whole entire career. The amount of open positions that we have and are unable to fill for different reasons, and now there really just aren’t bodies out there. This is even different than a nursing shortage because I think there’s nurses out there, but a lot of them have thrown in the towel.” (home health care, dyad 2)
Staffing - Facilitator
“A lot of hospital discharges historically start to happen- Thursday, Friday. Which means, we have an abundance of patients that need to be seen to meet that 48-h mark Saturday and Sunday. So, we have also launched several specific positions that are weekend resource positions so that we have more clinicians working some mixture where they- it encompasses a Saturday and Sunday. So it could be like a Thursday, Friday, Saturday, Sunday, they could be working for 10-h shift, so we got a little bit creative with that too. And we have hired in to more of that weekend position.” (home health care, dyad 2).
Access to care - Facilitator
“There are patients that are coming out of the hospital and perhaps they do not have the appropriate insurance or maybe they do not even have any coverage to begin with, so [HHC agency name] will do a courtesy visit, a one time charity visit, because the physician knows that this patient needs to be seen on home health. Then they’ll coordinate with us [outpatient transitional care] to make sure that we do the legwork on the insurance piece of it, or whatever the missing pieces are. They need a primary to sign off on it, etc., and then we will bridge that to help get that patient back to home health services within the week.” (outpatient, dyad 3)
Objective 7: Make a second nursing visit the first week of home health care
Access to Care - Barriers
“[name of a state] is a problem because [dyad 1] does not go to [X state]. And [name of HHC agency] is like the biggest home care agency there. And if the patient is not connected to them, they do not get- they will not take them. So that- and they come right out and say, ‘We’re not going to service it’. And that’s for all patients. So, it does not matter, their need.” (home health care, dyad 1)
“Yeah, so there’s some issues with some of the area hospitals. You know, they are kind of being like up for sale or whatever. And I feel like we are getting more and more patients that would have normally gone to [name of hospital] or [name of hospital]. And now they are coming to [our hospital], which is great for us. But now it’s even further away that these people, you know, patients are coming. So trying to get services in their area is tricky.” (home health care, dyad 1)
“Physical therapists, occupational therapists, speech therapists— they are allowed to go out, but for a nursing visit if they are heading to the physician then the nurse, cannot go.” (home health, dyad 2).
Home care internal policies, procedures, and pathways - Facilitators
“You know, one of the strategies that we have done with other patients, like, for example, wound care of that come through, wound care referrals; the schedulers will automatically schedule the start. And then a next day revisit. Because we know if the patient’s coming in for daily wound care, they are not only going to need to start, they are going to need a next day revisit. So instead of waiting until the clinician is home in an area where they can sync- sync meaning they put in their information, and then it pushes through so it’s transparent to everyone. Instead of being able to schedule, or not knowing that they need to get this patient covered for next day revisit, the schedulers will sometimes schedule things where they’ll already add to start, and then the next three is as needed right off the bat.” (home health, dyad 1)
“So for the nurses it’s similar. They assess the patient, they establish an appropriate visit order string that they discussed with the patient, and that’s appropriate based on the patient’s needs. And then, what they do is- they call ‘bulk schedule’ for the entire certification period. So if a patient requires wound care three times a week, they will bulk schedule the patient out three times a week. Sometimes it has to be like a Monday, Wednesday, Friday - it’s a wound vac type of dressing for the entire certification period.” (home health care, dyad 1)
Objective 8: Encourage and assist the patient to attend the outpatient visit
Patient education - Barriers
“We are finding a tremendous mismatch between what they were told in the hospital, which was kind of that default sentence of you know, ‘one or two weeks follow up with your primary care provider,’ and it was hard for us on the outpatient side to make these phone calls to patients who are saying ‘oh I’m not coming in, they told me I did not need to be seen for two weeks.’ (outpatient, dyad 1)
Competing priorities-social determinants of health - Barrier
“Well, you know it happens frequently that there are barriers. You know it’s the same kind of barriers that we see with any other appointment is ‘Am I going to have to pay something out of my pocket, can I afford it, does it rise up in the priority level in my own brain, do I have transportation, are there food insecurities and housing insecurities that kind of take precedence over whether or not I get to my appointments” (outpatient, dyad 5)
Access to Care - telemedicine - Facilitators
“Well, typically, it’s on the AVS [after visit summary], and so we can see it at discharge, and then when our nurses are planning their visits, they can coordinate either for that timeframe. Most often what we are seeing this happen for is like specialists or um I would say we have done some wound care visits this way, so when patients were terrified of going into the wound clinics, because of the Covid situation our nurses would be there for their Tele-health visit and then they would help show what the wounds look like on video, and then they would just take orders directly from the wound specialists at that time.” (home health, dyad 5)
“That’s my understanding and impression that people were happy to engage and, you know, more apt to engage in their [transition of care] (TOC) visit doing it virtually.” (acute care, dyad 1)

### 3.4 Barrier themes and (sub-themes)

*Care coordination* became difficult if the sepsis diagnosis was not clearly documented in the medical record. This arose when sepsis was either not diagnosed, not documented, or was resolved at discharge and fell to the medical history, rather than carrying forward as a condition requiring ongoing care. Multi-morbidity added complexity to the focus of care and there was variation in the operational definition of sepsis [Systemic Inflammatory Response Syndrome (SIRS), Sequential Organ Failure Assessment (SOFA)], Centers for Medicare and Medicaid Sep-1, which ascribes to Sepsis 2 definition, or Sepsis-3 (11, 33). When discussing how sepsis is documented in the EHR, barriers included competing diagnoses, difficulty identifying or over-identifying sepsis patients, and failing to use the word “sepsis.” Inconsistent *use of the problem list* in the *EHR* was a barrier to identifying sepsis with variation on how (e.g., sepsis as a resolved problem), or if the problem list was used at all. This had a cascading effect on *information transfer* to the next level of care and the ability to trigger the I-TRANSFER protocol.

*Care coordination* and *information transfer* posed challenges between acute and HHC, particularly with non-integrated EHRs and inconsistent or varied avenues for communication. Barriers included receiving too much or too little information, difficulty in finding sepsis diagnoses, and a lack of standardized communication. Post-acute HHC informants found it frustrating to receive multiple messages via texts, calls, or instant messaging, or to have no information at all. HHC informants noted they could not document sepsis if labeled resolved at discharge. *Scheduling* the first HHC visit and outpatient appointment before discharge and ensuring attendance were seen as *care coordination* and *staffing challenges*.

Informants unanimously supported the need for timely outpatient follow-up but identified a complex interplay of barriers to *scheduling* and *access to care* such as no one assigned to do it (*staffing*), where the patient lives and what takes precedence in their lives (g*eographic location, social determinants of health*), appointments made without patient or caregiver input (*provider or staff behavior*), scarce availability of open appointments or lack of access to outpatient schedules (*access to care*), conflicting information in discharge instructions (*patient education*), lack of transportation, and attempts to avoid co-pays (*competing priorities, financial insurance*). *Patient behavior, decisions, and preferences* to *decline or delay HHC,* not arrange or keep their outpatient appointments (*no-show outpatient*), prohibited timely care.

Staff shortages (*staffing*) in acute and HHC added to the challenges, particularly the ability to: find those who need HHC referrals, make outpatient appointments prior to discharge, and have enough HHC nurses to conduct the timely start of care or second visits. Severe *staffing* shortages and *geographic location* of the patient’s home affected whether a HHC agency accepted a patient.

### 3.5 Proposed strategies

Informants proposed strategies to overcome barriers to implementing the I-TRANSFER protocol. In ERIC taxonomy terms, some examples include *changing record systems* to create a banner or flag in the EHR to communicate sepsis survivors. The strategy to *facilitate relay of clinical data to providers* was often suggested in discussions of workflow in the home care referral and the discharge process. Other strategies suggested were to *use data warehousing techniques* to create reports to track patients throughout the transition *and intervening with patients to enhance uptake and adherence* through education, as well as *involve patients and family members* when making the outpatient appointment. Supplementary Table 2 reports proposed strategies, mapped to the ERIC taxonomy (23) and linked to the barrier theme and objective it addresses.

### 3.6 Facilitator themes

Prediction models and decision tree checklists within the *EHR* supported providers in identifying and diagnosing sepsis (*definition of sepsis*). The use of *EHR* add-on third-party software helped patients choose a quality HHC agency in their location. With the *use of the problem list,* the *EHR* facilitated *patient education* by populating education materials and documenting follow-up appointments in the after-visit summary (AVS) sent home with the patient. Integrated *EHR* systems facilitated *information transfer* across care settings. Software facilitating *information transfer* on the HHC referrals reduced *phone calls* and *messaging*. All HHC sites had at least a view into the acute care *EHR*.

*Quality monitoring of service,* including provider documentation, was a facilitator toward identifying the sepsis survivor in acute care. All sites had clinical documentation improvement (CDI) teams who monitored the EHR for signs and symptoms of sepsis (manually or via natural language processing) and queried providers to confirm or refute a sepsis diagnosis. The CDI team facilitated adding sepsis to the problem list, triggering daily reports to alert care coordinators, promoting *information transfer* to the next level of care, and prompting sepsis-specific *patient education.*

S*taffing,* in the form of full-time or part-time sepsis coordinators facilitated many of the I-TRANSFER objectives such as confirming the sepsis diagnoses, communicating sepsis to the team and patient, advocating and making post-acute care referrals, *patient education*, and *scheduling* outpatient appointments. One hospital used unit clerks to work with patients and caregivers to schedule outpatient appointments. One HHC agency reported increasing *staffing* on Friday–Sunday to handle surges in hospital discharges on Thursdays and Fridays. Sites also described *augmented services* (paramedic visits, home visiting providers, advanced illness management programs) to facilitate outpatient contact after discharge.

*Care coordination* was facilitated when the discharging provider signed the HHC orders for those without an *outpatient provider.* Few participating HHC agencies had liaisons in the hospitals as they were discontinued during the COVID-19 pandemic and not reinstituted, but when there was a HHC liaison on staff, they were described as a facilitator to make the HHC referral, schedule the first home visit, and coordinate services and equipment delivery. Acute care coordinators (*staffing)* assess discharge planning needs on each patient, and all sites had them (*acute care policies, pathways, and processes*).

*Access to care* was facilitated at sites with outpatient coordinators or in sites involved in the bundled payment program (*external policies and incentives*) because they are incentivized to increase quality and care coordination for 90-days. Designated personnel were responsible for ensuring HHC started after discharge and confirming patients had their medications, outpatient appointments, and transportation. Having Medicare insurance was another *access to care* facilitator and *telemedicine* emerged as a solution for outpatient appointments. Hospital at Home provider visits facilitated outpatient follow-up for the homebound (*augmented services*).

The presence of an *informal caregiver* facilitated sepsis education and the importance of timely post-acute attention. Informants mentioned calling from the bedside and using Facetime to educate caregivers.

*Home health internal policies, pathways, and processes* were compatible with I-TRANSFER HHC objectives. All the HHC leaders explained that ongoing efforts to comply with Medicare standards for the timely start of HHC and industry conventions for frontloading visits would facilitate the I-TRANSFER protocol.

## 4 Discussion

Barriers, proposed strategies, and facilitators were revealed during interviews to form 32 themes. *Care coordination* and *staffing* were the most saturated themes for barriers as they affected all eight I-TRANSFER objectives. Inadequate *staffing* for necessary tasks or visits was pervasive across all settings and sites, while having a sepsis coordinator or HHC liaison in place was advantageous. *Care coordination and access to care* themes contained barriers to getting an appointment and making and keeping the outpatient appointment within 7 days. The *EHR* was both a barrier and a facilitator for identifying sepsis, communicating sepsis, and coordinating sepsis care. The task of *information transfer* across settings faced several barriers but also had many facilitators in place and strategies for improvements were proposed. *Home care internal policies, processes and pathways* were compatible with I-TRANSFER in promoting the timely start of care and first week nursing visits. We situate our findings in relation to the literature and guidance from the Hospital Sepsis Program Core Elements (20).

To activate any intervention for sepsis we must first accurately identify and adequately document the sepsis diagnosis; in line with our objective 1. Establishing a standard definition of sepsis such as Sep-3 recommended by the Society of Critical Care Medicine and the European Society of Intensive Medicine (11) could help to address the serious information transfer and sepsis surveillance issues revealed by our study and others (34). The CDC Core Elements recommend standardized sepsis definitions, templated notes to document sepsis diagnosis and treatment, and inter-facility infection control transfer forms as processes to assure safe patient transfer (20). Facilitators present in our sites and the literature such as EHR algorithms, alerts, checklists, screening tools, and NLP are helpful to detect sepsis, but more work is needed to improve their accuracy and gain trust (35), reduce alert fatigue (36), and ensure their timeliness in the workflow (37).

A scoping review of 21 care transition intervention studies revealed parallels to our findings (1). Disrupted information flow negatively impacted care coordination and the transfer of information (4, 38, 39). Like other studies, our assessment found inconsistent use of the problem list (40, 41). This affected the ability to flag sepsis survivors for I-TRANSFER and ensure patients, informal caregivers, and providers were aware of the patient’s risk for rehospitalization and new or recurrent infection (42). Furthermore, identifying survivors in HHC was impeded by coding practices. HHC Coders were reluctant to place an acute care diagnosis of sepsis on the patient record if marked as resolved or not clearly documented as the reason for HHC. They reported they had no other code to use, so they labeled sepsis survivors as “other aftercare.” Based on this discovery, our team successfully petitioned the Centers for Disease Control for a post-acute ICD-10 code *Z51. A Encounter for Sepsis Aftercare* (43, 44). Acute care providers should optimize use of the problem list and educate teams to include the new aftercare code in discharge documents to enhance communication about sepsis survivorship and as a strategy to reconcile the effect of sepsis being reported as resolved on the problem list when aftercare is warranted to manage or rehabilitate new, lingering, or worsening sepsis-related problems. Incorporating this code in discharge documents can support improved information transfer and help identify sepsis survivors and trigger timely, sepsis focused interventions during the critical recovery period (our objectives 4, 6, 7, 8). Using the new code in post-acute settings will improve surveillance and tracking of sepsis survivors, enabling longitudinal study of sepsis survivors across care settings and time. This will deepen our understanding of the true burden of sepsis (34).

Our work reports barriers to optimal sepsis care in all six dimensions suggested by Draeger and colleagues (45) in their systemized review of 50 sepsis studies. Transitioning sepsis survivors for timely HHC and outpatient care is a complex process that requires communication, collaboration, and coordination across settings. One study identified 31 tasks associated with just the HHC referral process placing a burden on both acute and home health care staff (46). Staffing barriers included short staffing (47, 48), lack of dedicated staff (4), no sepsis coordinator or personnel to make outpatient appointments, and overwhelmed care managers (49, 50). The Hospital at Home program similarly faced care coordination and information transfer challenges due to non-integrated EHR’s (4).

Multiple care transition studies report the role of a transition nurse, social worker, coach, or care coordinator as a facilitator (1, 48, 51). In our study, sites with a sepsis coordinator identified them as a strong facilitator for implementing I-TRANSFER objectives, especially objectives 2–5 (identifying and referring patients for HHC, transferring information, making appointments). Sepsis coordinators could also facilitate implementation of the sepsis aftercare code, for the aforementioned reasons. In a pre-post evaluation of 13,877 patients, a multi-level quality improvement program with a sepsis coordinator proved sepsis coordinators cost effective with decreased mortality and length of stay (52). A systematic review of studies focused on the care transition experiences of patients, family members, and health care professionals reported seven out of 12 studies found that having an experienced case manager coordinating care facilitated intervention success (53).

An unexpected finding was how home health care internal policies, pathways, and processes were powerful facilitators of timely home health admission (our objective 6). The HHC industry standard to frontload visits for high-risk populations (54) and Medicare policies for a timely start of care within 2 days of discharge align with I-TRANSFER objectives. The Care Transitions Framework (CTF) (55) also supports these findings providing constructs for implementing care transitions innovations. Further, the Organizational Readiness to Implement Change survey of our sites revealed that HHC agency implementors were significantly more ready than acute care, perhaps due to existing policies and processes (56).

The Comprehensive Post-Acute Stroke Services Study (COMPASS-TC), which tested a hospital-to-home and outpatient care transitional care model (3), reported similar barriers to us including difficulty identifying eligible patients, insufficient staffing, lack of tracking systems/reports, clinic no-shows, and limited appointment availability (3). Insufficient insurance coverage and competing priorities like food and utilities may prevent patients from accepting home health or outpatient services due to co-pays. Screening for social determinants of health is recommended to identify and address these barriers (20). Addressing patient behavior, decision-making, and education is critical, as those unaware of, or unwilling to acknowledge their care needs may decline services and not seek care when needed (39, 51).

The CDC core elements emphasize communicating the sepsis diagnosis to primary care; we recommend expanding this communication to all post-acute settings, as well as to patients and caregivers. Diagnostic disclosure and patient education are essential- nearly half of sepsis survivors in a 2015 study were unaware of their diagnosis and associated risks (57). More research is needed that focuses on the educational and social needs of the patients and caregivers.

Outpatient care coordination and transition programs that contact patients after discharge were common facilitators at our sites. Home health clinicians followed established policies to ask about follow-up appointments and transportation, and they assisted with telemedicine visits when needed. However, for interventions like I-TRANSFER, calling within a week after discharge will be too late to ensure timely start of HHC or attendance at outpatient appointments. Our previous national study of sepsis survivors in HHC found that only 11% received outpatient visits within 1 week of discharge (19), compared to the AVENIR cohort in Germany where 68.8% of sepsis survivors saw a general practitioner within 2 days and 80% within 4 days (58). These comparisons highlight the urgent need to accelerate outpatient care timelines for sepsis survivors in the United States.

Although very challenging, care coordination interventions and protocols with multiple approaches to care transitions for sepsis survivors have resulted in improved rates of mortality, readmission, long-term physical function, and post-traumatic stress disorder symptoms (59). Aiming for similar results with the I-TRANSFER protocol, our next steps are to conduct and evaluate the implementation. We will perform implementation mapping (60) with the stakeholders to leverage the facilitators, operationalize the proposed strategies, and develop additional strategies to address the barriers we identified as implementation proceeds. During the implementation we will track the strategies implemented and quantify the effect of the implementation on readmission, ED use, and timeliness of care (18).

### 4.1 Limitations

The study is limited to five health systems (16 hospitals) and five HHC agencies in the northeast and western regions of the United States. The perspectives do not include sepsis survivors or their caregivers. Also, this analysis does not examine the relationship among specific sites, informant characteristics, and the determinants discussed. Future work will include more in-depth descriptions of certain themes and subthemes, analysis of the relationships between site characteristics, significant events affecting implementation, and the effects of the implementation on 30-day readmission.

## 5 Conclusion

A comprehensive needs assessment with 91 informants from acute, home health, and outpatient care revealed 32 themes containing barriers, proposed strategies, and facilitators critical to understand and address prior to implementation of a timely HHC and outpatient visit protocol for sepsis survivors. The interviews produced actionable insights for leaders, clinicians, providers, and policy makers regarding identifying sepsis through clear definitions, using the problem list and ICD-10 coding. Scheduling outpatient care, communicating to the next level of care, and providing timely follow-up and care coordination necessitates attention to staffing, tools for scheduling and quality measurement, and EHR integration for information transfer. Patient education is critical for awareness of risk and informed decision-making regarding follow-up after discharge.

## Data Availability

The data that support the findings of this study are available from the corresponding author upon reasonable request.
