# Supplementary material for: Context and determinants for implementing a sepsis survivor care transition intervention reported from five health systems and home health agencies
Source: Front Med (Lausanne). 2025 Dec 1;12:1632083. doi: 10.3389/fmed.2025.1632083 (PMC12702754; doi:10.3389/fmed.2025.1632083)
Supplement: Supplementary file 1 [file Supplementary_file_1.pdf]

Supplemental Table 1. Consolidated criteria for reporting qualitative studies (COREQ): 32-item checklist

No.	Item	Description	Section #
**Domain 1: Research team and reflexivity**			
Personal characteristics			
1.	Interviewer/facilitator	Which author/s conducted the interview or focus group?	page 3 line 248
2.	Credentials	What were the researcher's credentials? *E.g. PhD, MD*	title page
3.	Occupation	What was their occupation at the time of the study?	title page
4.	Gender	Was the researcher male or female?	n/a
5.	Experience and training	What experience or training did the researcher have?	title page
Relationship with participants			
6.	Relationship established	Was a relationship established prior to study commencement?	lines 235-236
7.	Participant knowledge of the interviewer	What did the participants know about the researcher? *E.g. Personal goals, reasons for doing the research*	lines 235
8.	Interviewer characteristics	What characteristics were reported about the interviewer/facilitator? *E.g. Bias, assumptions, reasons and interests in the research topic*	study PI and nurs[illegible]
**Domain 2: Study design**			
Theoretical framework			
9.	Methodological orientation and theory	What methodological orientation was stated to underpin the study? *E.g. grounded theory, discourse analysis, ethnography, phenomenology, content analysis*	lines 262-268
Participant selection			
10.	Sampling	How were participants selected? *E.g. purposive, convenience, consecutive, snowball*	lines 235-238
11.	Method of approach	How were participants approached? *E.g. face-to-face, telephone, mail, email*	lines 248-249
12.	Sample size	How many participants were in the study?	line 281
13.	Non-participation	How many people refused to participate or dropped out? What were the reasons for this?	none
Setting			
14.	Setting of data collection	Where was the data collected? *E.g. home, clinic, workplace*	workplace via zoo[illegible]
15.	Presence of non-participants	Was anyone else present besides the participants and researchers?	no

16.	Description of sample	What are the important characteristics of the sample? *E.g. demographic data, date*	table 1 and lines 2
Data collection			
17.	Interview guide	Were questions, prompts, guides provided by the authors? Was it pilot tested?	line 251 yes
18.	Repeat interviews	Were repeat interviews carried out? If yes, how many?	no
19.	Audio/visual recording	Did the research use audio or visual recording to collect the data?	yes both lines 251
20.	Field notes	Were field notes made during and/or after the interview or focus group?	yes lines 253-255
21.	Duration	What was the duration of the interviews or focus group?	one hour line 248
22.	Data saturation	Was data saturation discussed?	yes lines254-255
23.	Transcripts returned	Were transcripts returned to participants for comment and/or correction?	yes lines 270-271
**Domain 3: analysis and findings**			
Data analysis			
24.	Number of data coders	How many data coders coded the data?	8, lines 260-263
25.	Description of the coding tree	Did authors provide a description of the coding tree?	yes table 2
26.	Derivation of themes	Were themes identified in advance or derived from the data?	derived, line 267-2
27.	Software	What software, if applicable, was used to manage the data?	NVIVO 14 line 253
28.	Participant checking	Did participants provide feedback on the findings?	yes lines 270-271
Reporting			
29.	Quotations presented	Were participant quotations presented to illustrate the themes / findings? Was each quotation identified? *E.g. Participant number*	yes, tables 3, 4, ar
30.	Data and findings consistent	Was there consistency between the data presented and the findings?	yes, tables 3,4,and
31.	Clarity of major themes	Were major themes clearly presented in the findings?	yes, lines 260-398
32.	Clarity of minor themes	Is there a description of diverse cases or discussion of minor themes?	no
