# Supplementary material for: Context and determinants for implementing a sepsis survivor care transition intervention reported from five health systems and home health agencies
Source: Front Med (Lausanne). 2025 Dec 1;12:1632083. doi: 10.3389/fmed.2025.1632083 (PMC12702754; doi:10.3389/fmed.2025.1632083)
Supplement: Supplementary file 2 [file Supplementary_file_2.docx]

Supplemental Table 2. Example quotes of proposed strategies coded to the ERIC taxonomy, I-TRANSFER objective, and qualitative themes.

| **ERIC Implementation Strategy** | **Quote (Site)** | **Theme(s)** |
| --- | --- | --- |
| **Objective 1. Identify and document the sepsis diagnosis.** | | |
| Tailor strategies  Facilitate relay of clinical data to providers | In order to be deemed a sepsis survivor, we have to make sure we know that you had sepsis, so the problem is we're not always good about documenting someone is having sepsis in a way that is identifiable. What I mean by that is a provider may put it in a note, someone may state it, but if it's not added to a problem list then there's no trigger for someone to know that that person had sepsis and, as of now adding it to the problem list is the only way that it creates a transfer of this information right now. If there's an [automated process] where we lack it, it is the automated process to ensure that anyone leaving our health system has an automated way to say boom boom. This person is a sepsis survivor; that is what we lack. (dyad 1 acute care) | Themes: Information transfer; Electronic health record (unstructured documentation, structured documentation, use of the problem list) |
| Facilitate relay of clinical data to providers  Remind clinicians  Use data experts | Don’t you think we could have a colorful bar that says ’Sepsis Survivor’? I don’t know where that information pulls from or who needs to update it? (dyad 5 acute care) | Theme: Electronic health record (EHR alert) |
| Facilitate relay of clinical data to providers  Prepare consumers to be active participants  Involve patients/consumers and family members | Because not everybody [patient] is aware. …I just wish that there was a little bit more communication from the inpatient side letting us know “hey this patient, you know, is a sepsis patient. they're heading home”. Oh, then you call them, are you the one calling and sharing, or I don't know. I don't even know if any of that is even possible because it would take a lot of work. But it would really help if they knew, you know. (dyad 2 outpatient) | Themes: Information transfer; Electronic health record; Care coordination; Acute care policies, pathways, processes |
| Conduct ongoing training | Some of our difficulty is even with our home care visit where they're kind of coding them as pneumonia and UTI. I think sometimes we find that as a challenge as well. An easy place to look particularly on [EHR brand] would typically be a problem list. So, if sepsis is on the problem list that is one way that we typically do have identification. A lot of times, whoever, and some of this is really just playing with ongoing physician education and provider education is to try to use sepsis as the diagnosis it that's really the main diagnosis because we often see that a patient might be listed as UTI or pyelonephritis or pneumonia, bacteremia instead of really having that keyword sepsis. (dyad 2 home health care) | Themes: Electronic health record (structured documentation); Staff education; Provider or staff behaviors, decisions, preferences |
| Promote network weaving  Change record systems  Use data warehousing techniques | I mean I think care management is a, is definitely, you know, an absolute group that you need to partner with in this, because, maybe somehow they could generate a report from [EHR brand], I mean there's so many reports… you could utilize that flag, if it was activated in a formal way. (dyad 4 acute care) | Themes: Information transfer; Electronic health record (EHR alert) |
| Tailor strategies  Change record systems  Facilitate relay of clinical data to providers | We could probably similarly create a flag or something in the chart that says, this is a sepsis patient. I think that's the easy part if we can get our physicians to do it for you know correctly, because obviously they have to be the ones that identify the diagnosis right we can't as nurse case managers we can suggest. And we can say that that's what we think the patient should be labeled as, you know enable that for primary or principal diagnosis, but ultimately the physician has to say yes, this is the principal diagnosis. (dyad 4 acute care) | Theme: Electronic health record (EHR alert) |
| Tailor strategies  Facilitate relay of clinical data to providers | It really is truly confusing and ultimately, it will lead to different patients – it will lead to different definitions across campuses, so I think this idea of trying to come up with a shared understanding of what is sepsis is step one. Then I think that the problem list probably still should be the source of truth and some novel solution—it’s not that novel, but I do think figuring out a way to keep sepsis like, time and date stamp, that would be the ideal solution, and say sepsis, you know, I'll make it up—June 27 2021, and then if they click “resolved,” then the options would be “this was not sepsis,” then it disappears, or “sepsis now resolved,” and then it just goes to sepsis survivor, and then that would go in the AVS, and then, ideally—then it would be visible to the post-acute care world. (dyad 1 acute care) | Theme: Electronic health record (use the problem list) |
| Tailor strategies  Facilitate relay of clinical data to providers  Change record systems | Unless there's a, like you pointed out, an aftercare code, the codes that are available don't particularly point to sepsis. You know you can talk about weakness, you can talk about different aspects of recovery, renal insufficiency that kind of thing but that's not going to give you a sepsis, so you have to flag. (dyad 1 acute care) | Themes: Electronic health record (use of the problem list; EHR alert); Information transfer |
| Conduct education meetings  Facilitation  Promote network weaving  Conduct local needs assessment | Any of the information that you can send, would be great and what I can look at and through my  workflow and processes especially for my CDI [clinical documentation improvement] nurses, maybe identifying and bringing this kind of information to our sepsis group in the next month or so really identify maybe potential workflow opportunities. (dyad 5 acute care) | Theme: Staff education |
| **Objective 2: Identify sepsis patients for home care referral** | | |
| Facilitation  Involve patients and family members  Revise professional roles | I think having … a navigator, you know, certainly, having a navigator to help the patients through is what I would love to do for every disease process, because the patients love it, the families love it, you know, we have a navigator for cardiac, we have a navigator for breast, you know, they help the patients along with making the appointments, and they're just a resource for them to call if they have any questions, instead of necessarily calling their physician right away. (dyad 1 acute care) | Themes: Care coordination (scheduling); Staffing |
| Tailor strategies  Use data experts  Develop and implement tools for quality monitoring  Facilitation  Change record systems | We have that information in [EHR brand]. It is not something we normally track. We- we can access that information through the [EHR brand] system, but we do not- there's no formal tracking… But I would have to talk to the [EHR brand] team to see if there would be an easy way to track all patients. Right now, we don't have one in place. (dyad 1 acute care) | Themes: Electronic health record; Quality monitoring of service |
| **Objective 3. Refer patients to home health care** | | |
| Capture and share local knowledge  Conduct ongoing training  Promote network weaving | [Home health] liaisons aren't necessarily clinical, right? So, that's another piece. So, the education and training and standardization for me on those folks will be critical in order for us to, for them to understand why is this important and not to miss this, right? So, you know they're dealing with all sorts of data fields to come across demographic data fields, and if you're not clinical leaning, you're not aware that this is something that's important. It'll just be another thing that they either fill in, or they don't fill it, right? That, we've lost that on-site warm handoff, lots of those handoffs are occurring on zoom, and some of them are like SBAR hands-off, but to me that warm handoff from the bedside is missing. That's critical elements. (dyad 4 home health care) | Themes: Staff education; Home health agency policies, pathways, and processes; Provider or staff behavior, decisions, preferences |
| Tailor strategies  Facilitate relay of clinical data to providers | I think that there's opportunity to improve upon on more proactively identifying dates of discharge so that the home care team has more time to prepare and pick up the patient. We're not scrambling around that, you know, the 11th hour when the discharge order got written and it's six o'clock and they're finally leaving the campus, so. (dyad 2 home health care) | Themes: Information transfer; Care coordination (scheduling); Home health agency policies, pathways, and processes; Provider or staff behaviors, decisions, and preferences |
| Facilitation  Revise professional roles  Promote network weaving | We've got to get our arms around how to work in this environment… You would think, intake, we could be more productive not being on site, [but] it's not true it was much easier to walk in [the] room, interview the patient and family right there in place, be on the floor with the care managers, be able to go and see the physician, the nurse practitioners. [Now] everything is phone calls, phone calls, phone calls, nobody picking up it's very difficult. So, I believe there is a desire, but the environment is still very hard. (dyad 4 home healthcare) | Themes: Information transfer; Staffing; Care coordination; Provider or staff behaviors, decisions, and preferences |
| Identify and prepare champions | The engaged ambulatory clinic is part of us, there's a golden opportunity. All we have to do is pull up [EHR brand], press a button, and the referral comes over to us, and they can e-sign. However, to make that happen means that that project has to be elevated. It's been a year and a half, and it has sunk to the bottom throughout the entire Covid run, so it's played out. We know what needs to be done. (dyad 4 outpatient) | Themes: Electronic health record; Provider and staff behavior, decisions, and preferences |
| **Objective 4. Transfer clinical information to home health care** | | |
| Change record systems  Facilitate relay of clinical data to providers  Facilitation | I’ll talk to our social workers and case managers to see if it’s something down the road that we want to put in a free text field, there are things called smart phrases in [EHR brand] that we could click and put in, and we just have to talk to home care so we all know what that phrase means on their end as well, so if that's something we need to do, we can implement that here. (dyad 1 acute care) | Themes: EHR (structured documentation); Information transfer |
| Facilitate relay of clinical data to providers  Change record systems | ...So I think just trying to utilize that terminology a little bit more consistently um and try to find other ways through [EHR brand] that we might be able to find these patients a little bit earlier on this day or flag them. Um we've kind of talked about having either a banner or something along the lines of trying to flag a patient with sepsis so that it's very visible um in just the main screen of the chart where you're not having to kind of dig into either the problem list or a note um but that becomes a little challenging to you because it would either have to be something that's kind of manually added or you would have to use certain things like our predictive model and other tools that we've been developing to, you know, try to have that as a flag. (dyad 2 acute care) | Themes: Electronic health record; Care coordination (scheduling); Acute care policies, pathways, and processes |
| Facilitate relay of clinical data to providers  Tailor strategies  Change record systems  Revise professional roles | ...The bottom of our discharge summaries, we have to say, like, are they going home with services? So I wonder if it'd be easy enough to build in if you click home with services, and then another drop down menu comes down, were they septic in the hospital you click yes, and it just imprints that line you just said. That would only be like one extra click for a resident or hospitalist, if that line really makes such a big difference. (dyad 4 acute care) | Themes: Information transfer; Electronic health record (structured documentation) |
| Facilitate relay of clinical data to providers  Distribute educational materials | The only way that there's an automated communication is if on discharge, it says sepsis on the problem list, then this automates a trigger into our discharge summary, educational materials pump out to the patient, and then it's very clear on the discharge document only if it's on the problem [list] otherwise there is many other things that people are trying to do, but it's not like I said automated and 100% reliable. (dyad 1 acute care) | Themes: Information transfer; Staffing; Patient education; Electronic health record (use of the problem list) |
| Facilitate relay of clinical data to providers  Tailor strategies  Promote network weaving | We can still work a little bit better on transmitting information to non-[EHR brand] users that they have sepsis, you know. Just documents and warm handoff kind of a thing. (Dyad 1 acute care) | Themes: Electronic health record; Information transfer; Acute care policies, pathways, processes, Care coordination |
| Facilitate relay of clinical data to providers  Promote network weaving  Tailor strategies | … so maybe a provider gets notified of the discharge or issues that the patients have but maybe they don't forward it to anybody else to see or maybe the staff in the practice doesn't know that a patient was even in the hospital. Now, I think we have all definitely an opportunity to collaborate. (dyad 1 acute care) | Themes: Information transfer; Staffing; Care coordination; Provider or staff behaviors, decisions, preferences |
| Facilitate relay of clinical data to providers  Change record systems  Promote adaptability | When they’re discharged, it'll say you know discharged home with [name of agency] and then [the] home care documents in a different system that we have a read only access to. They do not have access to [EHR brand] so just to take you back to that one point… that they may not have the accurate data to support the correct diagnosis. That happens a lot yeah…I wish, they, homecare, had the same access to [EHR brand] as we do. I think that would be better communication across the board if they saw inpatient charts. Inpatient saw outpatient homecare chart. Right now, that's not there, I don't know when that will come up. I think, if that was resolved, we would have a lot of these gaps closed. (dyad 2 acute care) | Themes: Information transfer; Electronic health record; Scheduling |
| Facilitate relay of clinical data to providers  Change record systems | So, I guess if sepsis is ever added to the hospital list, I'm sure there's a way that on that post-acute care transfer report or something that it can be just carried through. (dyad 4 acute care) | Themes: Information transfer; Electronic health record (use of the problem list) |
| **Objective 5. Make the outpatient follow-up appointment** | | |
| Centralize technical equipment | Ideally if we could have a centralized way to do this [scheduling] it would be lovely. (dyad 1 acute care) | Theme: Care coordination (scheduling) |
| Facilitation  Inform local opinion leaders  Mandate change | I think somebody in administration needs to speak to the doctors to have them be open to seeing these patients in a timely manner. (dyad 1 acute care) | Theme: Care coordination (scheduling) |
| Develop resource sharing agreements  Identify and prepare champions  Tailor strategies  Revise professional roles  Promote network weaving | I would like that inpatient team to be able to just go in and book that appointment. Now again if the inpatient team goes in to do that and they can't find an appointment that they believe is within a reasonable timeframe. I absolutely want them to pick up the phone and phone a friend and say I need your help in making this happen. Right, whether that be our practice managers or a point of contact in our ambulatory care management team that might be able to help facilitate that but ideally they'd be able to go in and we have structured our scheduling templates in a way that would identify a TCM appointment within a reasonable amount of time. (dyad 2 outpatient). | Themes: Care Coordination (scheduling); Electronic Health Record; Information transfer; Staffing |
| Facilitation  Involve patients/consumers and family members | … I don't know if we do that for all patients, but I know that they had something happening, quite a few years ago where they were, you know, before the patient was discharged they would make sure that they, whether it was with the PCP or whatever, that they coordinated a first, at least appointment after discharge… Umm.. I guess that you'd have to look at a lot of the social and economic issues with different patients to see whether or not there’d be a reason why they couldn't make it to a doctor's appointment, whether or not they didn't have the transportation, or whatever, you know? And at least try to make it that their doctors appointment with somewhere that was close enough for them to get to, because you know, we're city…a hospital where a lot of our patients aren't really from right around here and setting up an appointment for a follow up for a patient that's actually, you know, still recovering, if they would have to come back into the city and take a two hour drive or something may not be in a rush to do it, you know? (dyad 4 acute care) | Themes: Care coordination (scheduling); Access to care; Competing priorities (social determinants of health) |
| Revise professional roles  Hire dedicated staff | But I think…in a team-based model we ought to have the ability to specialize a bit and to have dedicated people responsible [to schedule appointments]. I didn't mean that we have to ask our current social workers to make those appointments, and I completely agree they're spread thin. So you know the health system could invest in that resource…something we recommend. (dyad 1 acute care) | Themes: Staffing; Care coordination (scheduling) |
| Facilitation  Revise professional roles  Hire dedicated staff  Promote network weaving | …A lot of the smaller general practices that are independent from a health system do not allow you to make an appointment for a patient. They'll call you; you know you say you want to [help] with the patients being discharged, we'd like to send you the information and they said we'll call the patient back. So I’ve been…trying to maneuver my way in there as well, to try and get them to give me a date…I have just kind of continued maneuvering, yeah kind of pushing the ball [to] keep [it] going, but you can see where this is requiring a dedicated case manager…(dyad 1 acute care) | Themes: Care Coordination (scheduling); Staffing; Access to care (outpatient provider) |
| Revise professional roles  Involve patients and family members  Facilitation | We do so many admissions Thursday, Friday Saturday, Sunday, which is just another barrier because, you know, what we think really should happen for these patients is that the case manager should do the start, their case managers should be the one that's making sure they have the appointment. If you hand hold them then they'll do better, so I really think what needs to happen (dyad 2 home health) | Theme: Care Coordination (scheduling) |
| **Objective 6. Complete start of care visit within 48 hours of hospital discharge** | | |
| Involve patients and family members  Intervene with patients to enhance uptake and adherence  Tailor strategies | I think it'd be great to have a significant other present too, to understand what's going on, because, first of all, when you're sick and you've been through all that the hospital, you're only half hearing anything anybody's telling you anyway, you know, I think you need somebody else. I think for this to be successful, I would think you need to do this education and training, and have present at the time, somebody else that's going to reinforce it with the patient when they get home. I think they're going to see the benefit of it. I actually think the family or the significant other, whoever that is, is going to probably have a lot of benefit too because they're going to have to be dealing with what's happening at home, when things, when things happen. (dyad 1 home care) | Themes: Informal caregiving; Patient education |
| Conduct local needs assessment  Conduct local consensus discussions | Did you, you know, if the patient didn't have an appointment, you know what did you do, or just walk through the steps to figure that out, I mean, I think the first step would be maybe kind of doing you know some case reviews and interviewing some staff to figure out what kind of barriers might exist, but we haven't gotten that granular yet. (dyad 4 home health) | Themes: Care coordination (scheduling) |
| Facilitate relay of clinical data to providers  Develop and implement tools for quality monitoring  Promote network weaving  Change record systems | I agree that a care type is good because it is a flag. The care type will help for recording metrics purposes as well we can see if we run a report based upon sepsis you could see what was the readmission rate, how many patients came on service, that had a diagnosis of sepsis. So, from intake perspective, yes, it's important and then also for operations, I guess, it will trigger a pathway, a clinical pathway, on there about how the visit should be structured. That would have to probably be built in by the operations team. So…if they see that this patient had sepsis, this is what needs to happen so whether or not it's …embedded in home care home base, or if it's kind of like an educational coaching piece done on operation side to say okay if nurses who see that this is a sepsis patient, this is what you need to do. (dyad 4 home care) | Themes: Electronic health record (EHR alert); Quality monitoring; Home health agency policies, pathways, processes |
| Facilitate relay of clinical data to providers  Change record systems | The patient acuity is related to the hierarchy for visits…whether or not you know it does in terms of like triage and, if you think of that you know, like priority one, two and three…looking to add another level of acuity where it's a high-risk patient that requires the patients to be seen, like immediately or first visit or something like that, so that discussion actually started this week about looking to see about adding another acuity descriptor…for the sepsis patient and, if we look at it, that they are high risk that that would make sense that that acuity level is also aligned with those patients that require "you need to get in for this visit." Right now, you can only add it to care type because the second level of acuity that I was talking about, in terms of high-risk priority visit doesn't exist today. (dyad 4 home care) | Themes: Care Coordination (scheduling); Electronic health record (EHR alert) |
| Facilitation  Tailor strategies | I think, the quicker, we can get you know even just homecare involved, I think, is a big deal. Just because sometimes that we're calling patients on day one, two, or three, and we find discrepancies, where we need somebody to put eyes on them, and nobody else has been there yet. So even just that, alone, I think, would be huge if we can just guarantee that there's consistency for these home care nurses to be in the house, within a day or two of discharge. (dyad 2 home care) | Themes: Care coordination; Information transfer |
| **Objective 7. Make a second nursing visit that first week.** | | |
| Facilitation  Develop a formal implementation blueprint | …It's education. It might also just need to be clear clinical pathway related to sepsis patients, this is what you need to do, you know first visit A, B, C, D. Second visit A, B, C, D. Second visit within a seven-day window, within that five to seven day. You know that kind of thing. So, it's just very clear. It's prescribed. It's clear. It's standard practice. It's not left to folks deciding when and where to do it. (dyad 4 home care) | Themes: Staff education; Home health agency policies, pathways, processes |
| **Objective 8. Encourage and assist patients to attend outpatient visit.** | | |
| Tailor strategies  Promote adaptability  Facilitation | In a post-COVID world, would a virtual visit count?...It just was a new thought to me…Some patients, again, because of our geography here, we don't have a great public transportation system. We have [transportation] for patients who are Medicaid and who are over 65, they can ride Rover if they don't have transportation. But, you know, you have to call 24 hours in advance to book your transportation with Rover. And then it can be a two-hour process to go five miles because they are taking other people other places. (dyad 1 acute care) | Access to care (transportation, geographic location); Informal caregiving; Competing priorities (social determinants of health) |
| Facilitation  Tailoring strategies  Revise professional roles | That is the short-range solution… That they [nurse practitioners] are the option. This role is embedded within the TCM clinic, and then more TCM clinics for patient population clinics are within our clinic, and then home health gathering the information and feeding it to the NP who can, you know, it was a huge, missed opportunity. (dyad 3 home care) | Themes: Access to care (augmented services, telemedicine) |
| Facilitation  Conduct educational sessions  Tailor strategies  Promote adaptability  Conduct local needs assessment | I think in order for this to be successful, we're going to have to do some education and put some things in place. I think we might want to consider identifying at the start of care who might have a barrier to being able to get there, i.e. transportation or something like that. That specifically related to their TOC [transition of care] appointment. And I think in general this is probably something we got to do any way to get to that TOC appointment. Because I'm going to be willing [to do appointment] with [telehealth], if need be. (dyad 1 acute care) | Themes: Staffing; Staff education; Access to care (transportation); Telemedicine |
| Facilitation  Create new clinical teams  Tailoring strategies  Facilitate relay of clinical information to providers | My nurses, my clinical managers do what's called a start of care review. Which is, we require every clinician to call in regarding when they do a start of care. And a clinical manager goes over with them to make sure we've done it- dotted our I's and crossed our T's. I think it would fit well into this that they would have that conversation if they notice that the patient is a sepsis survivor. That we would, at that point, have a conversation about when is the TOC and what are their plans on getting there. I think we could build that into our start of care reviews and be able to pull MSW [Master of Social Work] in quick enough at that point. Because that- those start of cares happened within 24 hours of an admission. So that would give us at least a little bit of lead time to have a social worker, maybe be able to coordinate that care and or maybe coordinate that telehealth visit if we need it. (dyad 1 home care) | Themes: Staffing; Care coordination (scheduling); Home health agency policies, pathways, processes |
| **Strategies that affect all objectives** | | |
| Intervene with patient to enhance uptake and adherence  Involve patients/consumers and family members | I think there needs to be, I know it takes a while to have this kind of conversation especially when you're super busy on your unit, but a conversation about sepsis and then nursing really reviewing over those discharge papers and the sepsis instructions with them. (dyad 1 acute care) | Theme: Patient education |
| Facilitate relay of clinical data to providers  Conduct educational meetings  Conduct ongoing training  Inform local opinion leaders  Promote network weaving | We have really gotten more skilled in this area due to COVID. Learning how to communicate to a system team of about 300 employees in our department. So, we have several ways that we can do that. The first, since we’ll be focusing just on the main campus, to utilize the directors and managers there. We could do additional training. We could look at [Participant 1’s] team to do additional training about sepsis and then explain any new things that we would like to put in put in place like a focus on making sure we are clearly communicating that this patient is a sepsis survivor and we are sending them to home health care. We use email. We use Microsoft Teams page for announcements and then virtual trainings. (dyad 5 acute care) | Themes: Staff education; staffing; acute care policies, pathways, processes; Information Transfer (messaging system) |
| Facilitate relay of clinical data to providers  Revise professional roles | you need an escalation path that is actionable order. So if someone is volume overloaded, then he needs to go to someone that can say, ‘Okay, well. Can we give a dose of IV Lasix?’ If you can't do that they kind of go back. And then the one other thing is just the escalation path with primary care docs may not be the best. And again, they don't have a lot of time to handle the complex patients. (dyad 1 home care) | Themes: Care coordination; Access to care (augmented services) |
| Involve patients and family members  Prepare patients/consumers to be active participants  Revise professional roles  Conduct ongoing training | I feel like the family, the caregivers, I think need to be educated, so whoever the patient is going to be with when they get home because, clearly, even with the home health going in, and hopefully they're going to have some really great education on, you know, identifying early sepsis and everything, but I think you know, the people that are home with these patients should have some kind of education about what to look for and when to be concerned, and you know, like this is the home health aide so our nurses aren't going to be coming in daily, or they take like two days a week or something, and you know, as you know, people can get sick really quickly. (dyad 4 acute care) | Theme: Patient Education; Informal caregiving |
| Build a coalition  Identify and prepare champions  Model and simulate change | That's what I’m kind of hoping happens because I've seen it happen, and I think if ICU gets on board and Dr. [XX] gets on board who is a leader, he's the head of the sepsis committee, he believes in this. His group gets on board… I have to think it will. It will trickle down somehow. (dyad 1 acute care) | Themes: Acute care policies, pathways, and processes; Provider or staff behaviors, decisions, preferences |
